# Supplementary material for: Altered predictive control during memory suppression in PTSD
Source: Nat Commun. 2022 Jun 8;13:3300. doi: 10.1038/s41467-022-30855-x (PMC9177681; doi:10.1038/s41467-022-30855-x)
Supplement: Supplementary file 1 — Supplementary Information [file 41467_2022_30855_MOESM1_ESM.pdf]

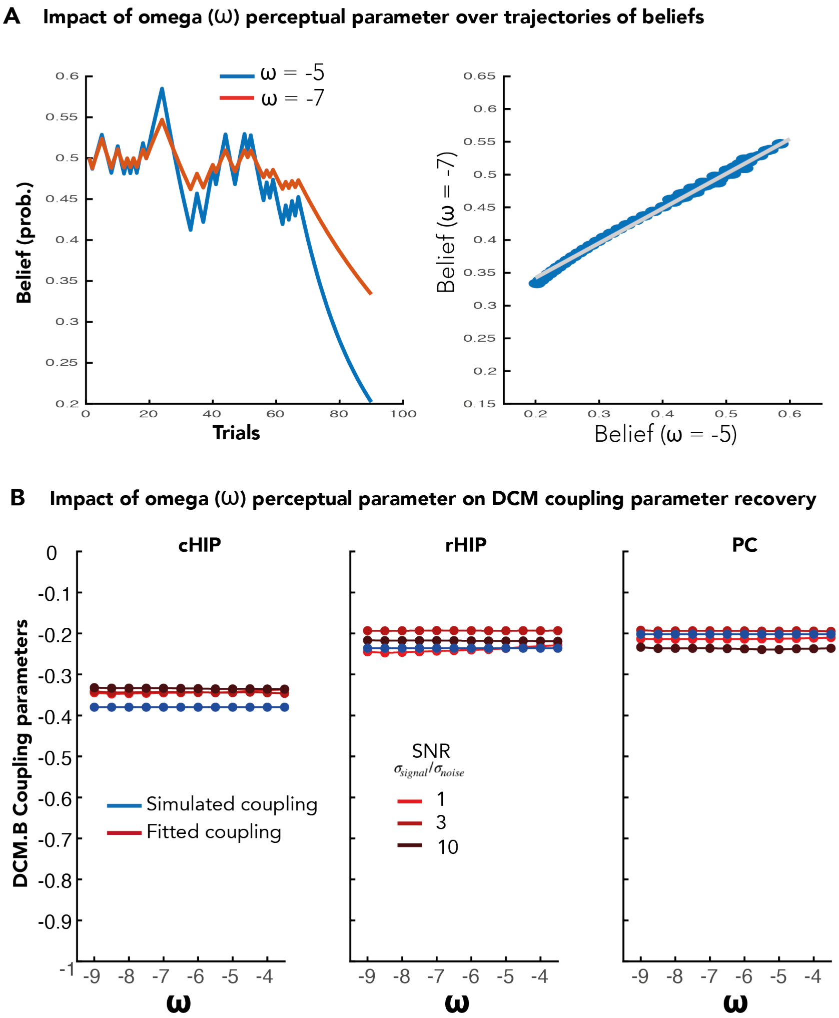

Supplementary Figure 1 – Influence of omega parameter on DCM coupling parameter recovery. (A) Illustration of the similarity between trajectories of belief with high (ω = -5) and low (ω = -7) perceptual parameter. (B) As a consequence, the ability to recover the true coupling parameter from generated synthetic data remains unchanged across ω levels. In this simulation, we simulated some fMRI data using fixed coupling parameters, but varying the belief trajectories according to different omega parameters. We then tried to recover the initial coupling parameter, and see whether the variation in the omega parameter has an impact on our ability to recover the true coupling parameter. Variations in ω have no impact of such DCM coupling parameter recovery. This is because the trial-to-trial variation of the belief that shapes the parametric modulation and influences the coupling parameter, remains largely identical after changing this perceptual parameter (see panel A). This plot shows the fitted coupling parameter, averaged across 2000 simulations conducted at three distinct Signal to Noise Ratio (SNR) levels.
