# Supplementary material for: Altered predictive control during memory suppression in PTSD
Source: Nat Commun. 2022 Jun 8;13:3300. doi: 10.1038/s41467-022-30855-x (PMC9177681; doi:10.1038/s41467-022-30855-x)
Supplement: Supplementary file 3 — Reporting Summary [file 41467_2022_30855_MOESM3_ESM.pdf]

nature research

Corresponding author(s): Pierre Gagnepain

Last updated by author(s): Mar 16, 2022

Reporting Summary

Nature Research wishes to improve the reproducibility of the work that we publish. This form provides structure for consistency and transparency in reporting. For further information on Nature Research policies, see our Editorial Policies and the Editorial Policy Checklist.

Statistics

For all statistical analyses, confirm that the following items are present in the figure legend, table legend, main text, or Methods section.

n/a	Confirmed	
☐	☒	The exact sample size (*n*) for each experimental group/condition, given as a discrete number and unit of measurement
☐	☒	A statement on whether measurements were taken from distinct samples or whether the same sample was measured repeatedly
☐	☒	The statistical test(s) used AND whether they are one- or two-sided<br>*Only common tests should be described solely by name; describe more complex techniques in the Methods section.*
☐	☒	A description of all covariates tested
☐	☒	A description of any assumptions or corrections, such as tests of normality and adjustment for multiple comparisons
☐	☒	A full description of the statistical parameters including central tendency (e.g. means) or other basic estimates (e.g. regression coefficient) AND variation (e.g. standard deviation) or associated estimates of uncertainty (e.g. confidence intervals)
☐	☒	For null hypothesis testing, the test statistic (e.g. *F*, *t*, *r*) with confidence intervals, effect sizes, degrees of freedom and *P* value noted<br>*Give P values as exact values whenever suitable.*
☐	☒	For Bayesian analysis, information on the choice of priors and Markov chain Monte Carlo settings
☐	☒	For hierarchical and complex designs, identification of the appropriate level for tests and full reporting of outcomes
☐	☒	Estimates of effect sizes (e.g. Cohen's *d*, Pearson's *r*), indicating how they were calculated

Our web collection on statistics for biologists contains articles on many of the points above.

Software and code

Policy information about availability of computer code

Data collection	All participants were scanned with a 3T Achieva MRI scanner (Philips) at the Cyceron Center (Caen, France).
Data analysis	Computational models were implemented in the TAPAS toolbox, version 3.0.0 (https://www.tnu.ethz.ch/de/software/tapas.html). Preprocessing of fMRI data and first-level DCM analysis were performed with SPM12 (https://www.fil.ion.ucl.ac.uk/spm/; version DCM12.5 revision 7479). The log-family evidence was computed using the MACS toolbox, version 1.3 (https://github.com/JoramSoch/MACS/releases/tag/v1.3), and Bayesian model comparisons were performed with the VBA toolbox (https://mbb-team.github.io/VBA-toolbox/). Codes for implementing model falsification, parameter and model recovery, as well as computational DCM, is available on GitHub (https://github.com/PierreGagnepain/predictive_control).

For manuscripts utilizing custom algorithms or software that are central to the research but not yet described in published literature, software must be made available to editors and reviewers. We strongly encourage code deposition in a community repository (e.g. GitHub). See the Nature Research guidelines for submitting code & software for further information.

Data

Policy information about availability of data

All manuscripts must include a data availability statement. This statement should provide the following information, where applicable:

- Accession codes, unique identifiers, or web links for publicly available datasets
- A list of figures that have associated raw data
- A description of any restrictions on data availability

All the raw behavioral and imaging data are archived at the GIP Cyceron center in Caen and are part of an ongoing longitudinal research project. Raw behavioral and brain imaging data are available under restricted access for the ethical restrictions for the current research project. These data can only be shared with researchers working on similar topics, upon reasonable request, via data request to Dr. Pierre Gagnepain (pierre.gagnepain@inserm.fr). The clinical data and the subject-specific

DCM coupling parameters data necessary for the statistical analyses of the current research article have been deposited on the GitHub repository (https://github.com/PierreGagnepain/predictive_control) and are also available on Zenodo (https://doi.org/10.5281/zenodo.6362400).

Field-specific reporting

Please select the one below that is the best fit for your research. If you are not sure, read the appropriate sections before making your selection.

☒ Life sciences ☐ Behavioural & social sciences ☐ Ecological, evolutionary & environmental sciences

For a reference copy of the document with all sections, see nature.com/documents/nr-reporting-summary-flat.pdf

Life sciences study design

All studies must disclose on these points even when the disclosure is negative.

Sample size	No statistical methods were used to pre-determine the sample size. However, the sample size used in this study for exposed participants was determine to ensure that the number of participants in both clinical group (PTSD- and PTSD+) commensurate or exceed previous studies investigating neurofunctionnal markers of memory control (with usually n = 20-30) . The sample size of the nonexposed group was determined to match aproximatively half of the exposed sample size. The initial sample size for each exposition group (nonexposed and exposed) was determined to satisfy the couterbalancing of material list across participants.
Data exclusions	In total, this multiwave multimodal brain imaging programm included 200 participants (120 trauma-exposed and 80 nonexposed). Data from eight nonexposed participants were excluded from further analyses for the following reasons: absence of intrusion rating owing to technical or behavioral issues (n = 5), artifacts in the MRI images (n = 2), and inability to pursue the experiment (n = 1). Data from 19 exposed participants were excluded from further analyses for the following reasons: absence of intrusion rating owing to technical or behavioral issues (n = 9), interruption of participation during the MRI acquisition (n = 3), and non-respect of diagnostic inclusion criteria (n = 7).
Replication	Given the unique and exceptional nature of this project, a replication study is not possible. However, longitudinal data have been collected and will permit to understand the stability, reliability, and evolulation of the neurofunctional markers at stake.
Randomization	This study includes three experimental groups : individuals exposed to the November 13th 2015 with or without PTSD, as well as non-exposed. Exposed participants were randomly recruited through a transdisciplinary and longitudinal research “Programme 13-Novembre” (www.memoire13novembre.fr/), as well as through victims’ associations.
Blinding	During data acquisition, investigators knew whether the participants were exposed or not to the attacks. For ethical (medical monitoring of traumatized indviduals) and practical (clinical interview and trauma-related questionaire) reasons, it was not possible to blind the exposed status of the participants to the experimentator. However, investigators were blind to PTSD diagnosis and had no a priori knowledge of group assignment which was determined after data acquisition.

Reporting for specific materials, systems and methods

We require information from authors about some types of materials, experimental systems and methods used in many studies. Here, indicate whether each material, system or method listed is relevant to your study. If you are not sure if a list item applies to your research, read the appropriate section before selecting a response.

Materials & experimental systems

n/a	Involved in the study	
☒	☐	Antibodies
☒	☐	Eukaryotic cell lines
☒	☐	Palaeontology and archaeology
☒	☐	Animals and other organisms
☐	☒	Human research participants
☐	☒	Clinical data
☒	☐	Dual use research of concern

Methods

n/a	Involved in the study	
☒	☐	ChIP-seq
☒	☐	Flow cytometry
☐	☒	MRI-based neuroimaging

Human research participants

Policy information about studies involving human research participants

Population characteristics	55 exposed participants had full or partial PTSD (+) + (30 females and 25 males, mean age = 37.14, SD = 8.35) and 46 did not (PTSD-, 16 females and 30 males, mean age = 36.84 years, SD = 7.05 years). This study also included 72 nonexposed participants (38 females and 34 males, age = 33.69 years, 33.66 SD = 11.40 years).
Recruitment	Exposed participants were recruited through a transdisciplinary and longitudinal research “Programme 13-Novembre” (http://www.memoire13novembre.fr/), as well as through victims’ associations. Nonexposed participants were

	recruited through our local panel of volunteers. No potential recruitment bias is likely to impact results to our knowledge.
Ethics oversight	The study was approved by the regional research ethic committee ("Comité de Protection des Personnes Nord-Ouest III", sponsor ID: C16-13, RCB ID: 2016-A00661-50).

Note that full information on the approval of the study protocol must also be provided in the manuscript.

Clinical data

Policy information about clinical studies

All manuscripts should comply with the ICMJE guidelines for publication of clinical research and a completed CONSORT checklist must be included with all submissions.

Clinical trial registration	clinicaltrial.gov registration number: NCT02810197
Study protocol	The study protocol was submitted to INSERM (sponsor ID: C16-13, RCB ID: 2016- A00661-50)
Data collection	The data were acquired between June 13th 2016 and June 7th 2017 (i.e., between seven and 18 months after the attacks, PTSD participants: mean (SD)=14.30 (3.28) months; Trauma-exposed non-PTSD group: mean (SD)=13.48 (3.08) months; group comparison: t(90)=1.20, p=0.22). Experiment took place in Centre Cyceron, Boulevard Becquerel, BP 5229, F-14074 Caen Cedex
Outcomes	As a primary outcome, exposed participants were diagnosed using the Structured Clinical Interview for DSM-5 (SCID, American Psychiatric Association, 2013) conducted by a trained psychologist and supervised by a psychiatrist. All exposed participants met DSM-5 criterion A indicating that they experienced a traumatic event. Trauma-exposed participants were divided into two groups: one with full or partial symptomology of PTSD (Brancu et al., 2016) according to DSM-5 criteria and one without PTSD. As a secondary outcome, participants performed the PTSD check list for DSM-5 (Blevins et al., 2015), as well as the Beck Depression Inventory (BDI) and the State-Trait Aniety Inventory (STAI) to measure the intensity of symptoms.

Magnetic resonance imaging

Experimental design

Design type	Event-related design
Design specifications	The TNT task was divided into four sessions of ~8 min each. In each session, the 18 think and 18 no-think items were presented twice. Word cues appeared for 3 s on the screen. After each trial, participants had up to 3600 ms to make the intrusion rating, followed by y a jittered fixation cross (1400, 1800, 2000, 2200, or 2600 ms)
Behavioral performance measures	Word cues appeared for 3 s on the screen and were written either in green for think trials or in red for no-think trials. After the end of each of the think or no-think trial cues, participants reported whether the associated object had entered awareness by pressing one of two buttons corresponding to "yes" (i.e., even if the associated object pops very briefly into their mind) or "no".

Acquisition

Imaging type(s)	functional
Field strength	3T
Sequence & imaging parameters	MRI data were acquired on a 3T Achieva scanner (Philips). All participants first underwent a high-resolution T1-weighted anatomical volume imaging using a 3D fast field echo (FFE) sequence (3D-T1-FFE sagittal; TR = 20 ms, TE = 4.6 ms, flip angle = 10°, SENSE factor = 2, 180 slices, 1 mm by 1 mm by 1 mm voxels, no gap, FoV = 256 mm by 256 mm by 180 mm, matrix = 256 by 130 by 180). This acquisition was followed by the TNT functional sessions and an eyes-closed resting-state fMRI sequence, which were acquired using an ascending T2-star EPI sequence (MS-T2-star-FFE-EPI axial; TR = 2050 ms, TE = 30 ms, flip angle = 78°, 32 slices, slice thickness = 3 mm, 0.75-mm gap, matrix 64 by 64 by 32, FoV = 192 mm by 192 mm by 119 mm, 235 volumes per run). Each of the TNT and resting-state functional sequence lasted about 8 min.
Area of acquisition	Whole-brain

Diffusion MRI ☐ Used ☒ Not used

Preprocessing

Preprocessing software	Image preprocessing was first conducted with the Statistical Parametric Mapping software (Statist Parametric Mapping software (SPM12, University College London, https://www.fil.ion.ucl.ac.uk/spm/) implemented on Matlab R2019a. Functional images were (i) spatially realigned to correct for motion (using a six parameter rigid body transformation); (ii) corrected for slice acquisition temporal delay; and (iii) co-registered with the skull-stripped structural T1 image. The T1 image was bias corrected and segmented using tissue probability maps for gray matter, white matter, and cerebrospinal fluid.
Normalization	The forward deformation field was derived from the nonlinear normalization of individual gray matter T1 images to the T1 template of the Montreal Neurological Institute (MNI). Each point in this deformation field is a mapping between MNI

	standard space to native-space coordinates in millimeters. Thus, this mapping was used to project the coordinates of the MNI standard space ROIs to the native space functional images.
Normalization template	MNI
Noise and artifact removal	The preprocessed fMRI time series at each voxel were high-pass filtered using a cutoff period of 128 s. Regressors of no interest were the six realignment parameters to account for linear residual motion artifacts. Temporal autocorrelation between the GLM residuals was corrected using the first order autoregressive process resulting in pre-whitened data after restricted maximum likehood estimation.
Volume censoring	We did not applied volume censoring.

Statistical modeling & inference

Model type and settings

1) We used computational modeling to estimate the formation of intrusive belief during the memory suppression. These models included 2 levels Hierchical Gaussian Filter, Rescorla-Wagner, and Kalman filter. Model falsification, as well as parameter and model recovery analyses were performed to validate the outcomes of this modelling effort (Wilson and Collins, 2019; Palminteri et al., 2017)

2) Beliefs and resulting prediction-error estimated at step 1 were used as parametric modulators of the inputs (i.e., stick function) modulating the top-down coupling between control and memory systems in DCM. DCM pathways were selecetd using Bayesian Model Selection and protected exceedence probabilities (PXP). DCM coupling parameters quantifying the effect of control (Beliefs and prediction-error) were estimated using Bayesian Model Averaging (BMA).

3) The effect of control was further analysed using a Balance Index. Belief and prediction-error control were projected as two directional forces on two distinct orthogonal axes (i.e. separated by a 90° angle) in a two-dimensional circular space. We fixed the 0° position on the south axis of the circle, and computed the direction of the resultant vector combining these two forces with respect to this optimally balanced position. From this ideally balanced 0° position, imbalance index is given by the angle of resultant vector of forces.

Effect(s) tested

Statistical analyses were performed on coupling parameters estimated after BMA, using one-tailed t tests according to a priori hypotheses, in the three target memory regions (rHIP, cHIP, PC), as well as the wHIP (i.e., four regions in total). Four effects were tested:
1) Control * Group interactions comparing the control effect (predictive - reactive) in PTSD+ with both PTSD- and nonexposed in all four regions (i.e., 8 tests in total):
2) Control effect (predictive - reactive) in all three groups and four regions (i.e., 12 tests in total);
3) Reactive negative coupling in all three groups and four regions (i.e., 12 tests in total);
4) Predictive negative coupling in all three groups and four regions (i.e., 12 tests in total).

For completeness, we also computed the Pp of the groups' coupling parameters, as well as the bootstrapped 95% CI of the mean.

In addition, we also report Bayes factors (BF) as effect size in Table 1, using a Markov chain Monte Carlo (MCMC) method64. BF represent the likelihood of suppression effects for each within-group comparison. Based on this hypothesis, we defined a region of practical equivalence (ROPE) set as a Cohen's d effect size greater than "0.1". The MCMC method generated 90,000 credible parameter combinations that are representative of the posterior distribution. Then, the BF was estimated as the ratio of the proportion of the posterior within the ROPE relative to the proportion of the prior within the ROPE. The conventional interpretation of the magnitude of the BF is that there is substantial evidence for the alternative hypothesis when the BF ranges from 3 to 10, strong evidence between 10 and 30, very strong evidence between 30 and 100, and decisive evidence above 100.

To further ensure the PTSD specificity of our findings, we tested Group*Control type interaction through ANCOVAs by controlling for three transdiagnostic anxiety-related dimensions (anxious arousal, dysphoric arousal and general anxiety), and three transdiagnostic affect-related dimensions (anhedonia, mood and depression).

Correlations between DCM parameters and symptoms were computed using robust skipped-Spearman correlation (Pernet et al., 2012)

In order to compare Balance Index between groups, we used Watson-Williams two-samples test, which approximates one-way ANOVA for circular statistics.

Specify type of analysis: ☐ Whole brain ☒ ROI-based ☐ Both

Anatomical location(s)	DCM entails a priori selection of regions of interest (ROIs).<br>The following ROIs included in the DCM models were initially selected from the Brainnetome atlas (BNA, http://atlas.brainnetome.org/):<br>- aMFG included both BNA's A46 (centre coordinates: x = 28, y = 55, z = 17) and A9/46v (centre coordinates: x = 42, y = 44, z = 14). We excluded voxels with y coordinates < 35 mm;<br>- pMFG included both BNA's A9/46d (centre coordinates: x = 30, y = 37, z = 36) and A8vl (centre coordinates: x = 42, y = 27, z = 39). We excluded voxels with y coordinates > 25 mm;<br>- rHIP (centre coordinates: x = 22, y = -12, z = -20);<br>- cHIP (centre coordinates: x = 29, y = -27, z = -10);<br>- PC included BNA's dmPOS (centre coordinates: x = 16, y = -64, z = 25).<br>The five ROIs were projected into participants' native space using the deformation field, without any spatial warping nor smoothing of the functional images, in order to ensure maximum accuracy.<br>Beta parameters for Think and No-Think conditions were estimated during a second-pass of the GLM using ordinary least-square and used to calculate subject-specific t-maps for each ROI. For each participant and ROI, we identified the maximum activation peak (using No-Think>Think contrast for aMFG and pMFG, and No-Think<Think contrast for memory regions) and the thirty most significant and contiguous voxels around that peak.
Statistic type for inference<br>(See Eklund et al. 2016)	The current study did not employ whole-brain statistical parametric map and therefore statistical inferences for spatial extent was not applicable.
Correction	The expected proportion of type I error across multiple testing was controlled using the False Discovery Rate (FDR) correction, with a desired FDR q = .05 and assuming a positive dependency between conditions.

Models & analysis

n/a	Involved in the study
☐	☒ Functional and/or effective connectivity
☒	☐ Graph analysis
☒	☐ Multivariate modeling or predictive analysis

Functional and/or effective connectivity	Model-based Dynamic Causal Modelling for fMRI
